# Stability and electronic structure of hydrogen vacancies in ADP: hybrid DFT with vdW correction

**DOI:** 10.1039/c7ra13212c

**Published:** 2018-02-12

**Authors:** Tingting Sui, Yafei Lian, Mingxia Xu, Lisong Zhang, Yanlu Li, Xian Zhao, Xun Sun

**Affiliations:** State Key Laboratory of Crystal Materials, Shandong University Jinan 250100 China liyanlu@sdu.edu.cn sunxun@sdu.edu.cn

## Abstract

The formation energies, charge transition levels, and electronic structures of positively charged, neutral, and negatively charged hydrogen vacancies in the NH_4_H_2_PO_4_ (ADP) crystal are investigated in the framework of density functional theory with local and hybrid exchange–correlation functionals. The inclusion of nonlocal exchange opens the ADP fundamental band gap by nearly 1 eV and well reproduces the experimental value. The van der Waals (vdW) interaction is found to have a major influence on the energetics of charged hydrogen vacancies in ADP. The calculated relative stability of 
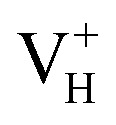
 and 
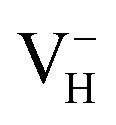
 with vdW interaction could well explain the break point on the measured conductivity curve of the ADP crystal in the high temperature region. On the other hand, a missing H atom in the (H_2_PO_4_)^−^ group is found to be more energetically preferable than NH_4_^+^. It could capture a hole carrier to form a molecular-type polaron with its adjacent two O atoms, and be responsible for the optical absorption under irradiation by a high-intensity laser beam.

## Abstract 2

Hybrid DFT calculations with vdW correction well explain the defect-induced conductivity break of ADP in the high-temperature region.

## Graphical abstract

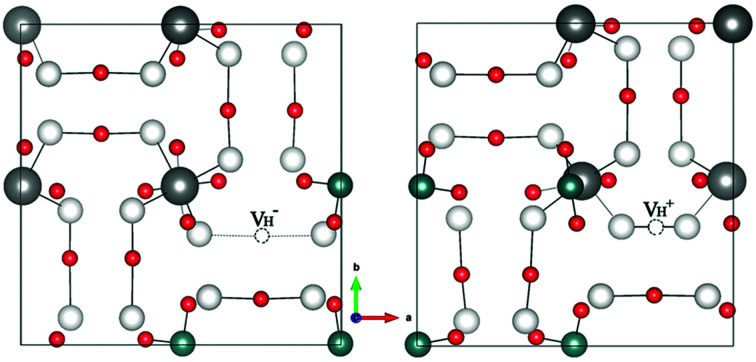

## 1. Introduction

The ammonium dihydrogen phosphate (NH_4_H_2_PO_4_, ADP) crystal is one of the promising materials exploited in nonlinear optical and electro-optic fields, which has been widely used in high power laser systems, realizing second, third and fourth harmonic generation.^1–4^ As compared to its analogue potassium dihydrogen phosphate (KH_2_PO_4_, KDP), ADP exhibits a higher laser damage threshold (LDT) and larger nonlinear optical (NLO) coefficient than KDP, and can thus be treated as a promising alternative material to break the present KDP monopoly of Inertial Confinement Fusion (ICF) applications.

Much experimental work has been reported on the crystal growth, morphology, structure analysis and the optical performance of ADP crystals.^5–13^ For example, the morphology of tetragonal bipyramid for ADP and its nature crystal structure have been defined by Xu Dongli *et al.*^14^ Moreover, Zhao *et al.* studied the crystallization mechanism of ADP, which was regarded as the process related to bond formation and breaking, connecting structure, growth morphology and microscopic chemical bonds.^15^ With *in situ* ATR-IR spectroscopy, the hydrogen bonding of NH_4_^+^ and (H_2_PO_4_)^−^ groups during ADP crystallization as well as the difference with that in its aqueous solution were reported recently.^16^ In 2016, series of ADP crystals have been grown under different experimental conditions and all the crystals were found to exhibit higher LDT than KDP.^4^ On the other hand, some theoretical work have also been done regarding to the chemical bonding, crystal structure, and physical properties of ADP crystals under ideal environment. For instance, the morphology of ADP crystals were modelled based on the calculation of chemical bond strength and the interaction between crystal surfaces.^17,18^ The density functional theory (DFT) calculation was carried out to present the microscopic mechanism of antiferroelectricity in ADP crystal.^19^

It is noted that the LDT of ADP could be reduced due to the existence of intrinsic point defects in the crystal. Many experimental efforts have been made to detect the defects in the crystal. In Murphy's research, there is an inflection point on the conductivity curve between the low and high temperature areas, which can be used to characterize the formation energy and diffusion of the defect.^20^ As Pollock didn't find such inflection point, he proposed that the defect formation and diffusion is a process in ADP crystal.^21^ Later, Harris proved that the proton vacancy in NH_4_^+^ contributes to the electric conduction of ADP.^22^ Rath further illustrated the impact of impurity and hydrogen vacancy on the conductivity and dielectric loss of doped ADP crystals.^23^ Also, Abdel-Kader regarded the inflection point as the relative rotary movement of phosphate group with its surrounding hydrogen proton.^24^ Therefore, hydrogen vacancy plays a key role in the physical properties of ADP such as electrical conductivity, dielectric loss, *etc.* Furthermore, the hydrogen vacancy could introduce impurity absorption that will reduce the optical quality of the crystal. It could also trap electron or hole carriers to bring out the change of electronic and optical properties. However, the influence of hydrogen vacancy defect on the physical properties of ADP crystals is still in dispute in experiment. The related theoretical studies have not been reported yet. Studying the structure and properties of hydrogen vacancy defect in ADP could help us to understand the microcosmic mechanism of defect induced conductivity and optical damage.

In this paper, we investigated the relative stability and electronic structures of charged hydrogen vacancy defects in ADP crystal by using hybrid DFT. The influence of supercell size, the electron exchange and correlation functional, and van der Waals (vdW) interaction on the defect formation energies of charged hydrogen vacancies are carefully examined in order to guarantee the accuracy of calculation results. Hybrid DFT was used to obtain more reliable description of defect formation energies, defect levels, and the localization of the electron distribution than (semi)local generalized gradient approximation (GGA) functional in wide-band-gap semiconductors^25–30^ by introducing some amount of exact exchange from Hartree–Fock theory in the electron exchange and correlation functional. The vdW interaction is found to be very important in describing the formation energies and defect levels for ADP crystal.

## 2. Computational method

The Vienna *Ab Initio* Simulation Package (VASP)^31,32^ based on the first-principles and the projector-augmented-wave (PAW) potential is implemented in the present calculation. Thereby the N 2s^2^2p^3^, H 1s^1^, P 3s^2^3p^3^, and O 2s^2^2p^4^ states are treated as valence electrons. The general gradient approximation (GGA) of Perdew Burke and Ernzerhof (PBE)^33^ is employed to optimize the crystal structure with the force convergence criterion of 0.01 eV Å^−1^. The energetics and electronic structures are calculated by the screened-exchange hybrid density functional of Heyd, Scuseria, and Ernzerhof HSE06 (ref. 34 and 35). In this approach, the long-range exchange potential and the correlation potential are calculated with PBE functional, while the short-range exchange potential is calculated by mixing a fraction of nonlocal Hartree–Fock exchange with PBE. The screening length and mixing parameter are fixed at 10 Å and 0.25 respectively. To confirm the convergence of the calculations, the dependence of the total energy on the *k*-point set mesh^37^ and cutoff energy of bulk ADP is investigated with PBE and HSE06 respectively.^36^ According to the convergence test, the cutoff energy is set to 400 eV for both PBE and HSE06 calculations, and the *k*-point sets are chosen to 4 × 4 × 4 and 2 × 2 × 2 for PBE and HSE06 calculations respectively. Another two types of hybrid functionals HSE03 (ref. 38) and PBE0 (ref. 39 and 40) are also used to test the performance of hybrid functionals on the defect formation energy and the electronic properties. The supercell containing 48 atoms are used to construct the defect models. All the atoms and the lattice constants are fully relaxed by using the conjugate gradient techniques. DFT/vdW-WF2 method^41–43^ is used to consider the hydrogen bond interaction in the whole system. The tetragonal supercell with four NH_4_H_2_PO_4_ units (48 atoms) is used to model hydrogen vacancy defects in the crystal with defect density of 2.08 atom%. An electron is removed or added to the neutral system of hydrogen vacancy 
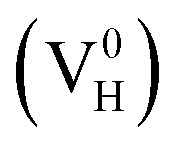
 in order to model the positively 
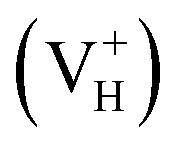
 and negatively 
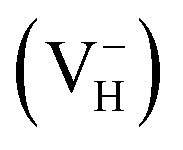
 charged hydrogen vacancy structure. The charged systems are also fully relaxed. The lattice vectors are refined as **A** = (*a***i** + *a***j**), **B** = (*a***i** − *a***j**), **C** = *c***k** with the lattice constants of conventional unit cell *a* = *b* = 7.50 Å, and *c* = 7.55 Å.^44,45^

The defect formation energies *E*_f_ of defect *X* with charge state *q* dependent on the Fermi level position is defined as^46–48^1
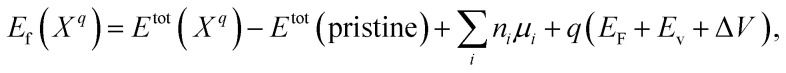
where *E*^tot^(*X*^*q*^) is the total energy of a supercell with defects. *E*^tot^(pristine) is the total energy of the supercell without any defects. *n*_*i*_ is the species number of the atoms which is removed or added to the supercell. *μ*_*i*_ is the chemical potential of the element *i*. *E*_F_ is the Fermi energy with respect to the valence-band maximum (VBM) *E*_v_ in the pristine single crystal. Δ*V* is the difference between the electrostatic potentials of the defective and pristine systems. The chemical potential of H is calculated by putting a H_2_ molecule in a cubic cell with lattice constant of 10.00 Å. The values calculated by many considered functionals and those considered van der Waals interactions are listed in Table 1. It is seen that the calculation values of H chemical potential mainly depend on the chosen of the type of the electron exchange–correlation potentials. The traditional PBE functional always overestimates the energy of H as compared to the hybrid functionals.

**Table 1:** The chemical potentials of H with different DFT functionals and van der Waals interaction

Functionals	The chemical potential of H (eV)
PBE	−3.46
HSE06	−3.82
HSE03	−3.82
PBE0	−3.95
PBE + vdW	−3.34
HSE06 + vdW	−3.82

The thermodynamic transition levels *ε*(*q*_1_/*q*_2_) is defined as the Fermi-level position for which the formation energies of charge states *q*_1_ and *q*_2_ are equal:^46,49^2
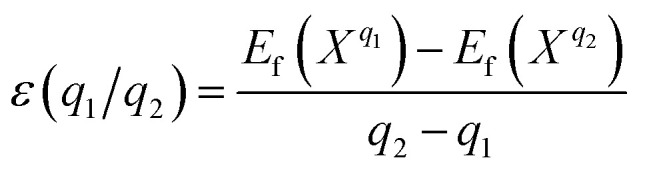
where *E*_f_(*X*^*q*^) is the formation energy of the defect *X* in the charge state *q*. The experimental significance of the charge transition level is that for Fermi-level position below *ε*(*q*_1_/*q*_2_), charge state *q*_1_ is stable, while for Fermi-level position above *ε*(*q*_1_/*q*_2_), charge state *q*_2_ is stable.

## 3. Results and discussion

### 3.1 The structure of hydrogen vacancies in ADP

**Fig. 1:**
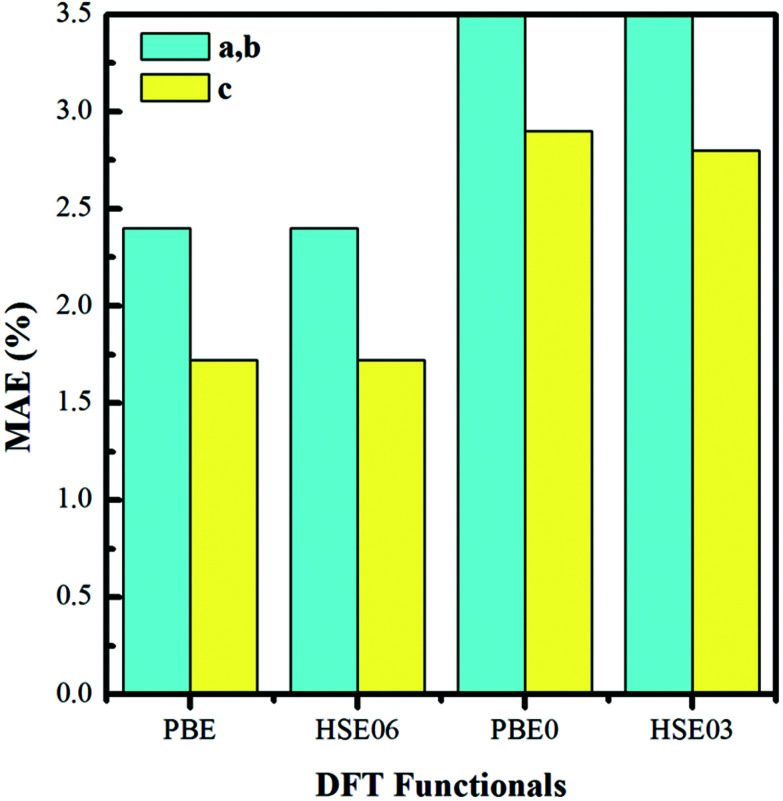
Mean absolute error (in %) on the optimized lattice constants of bulk ADP as compared with the experimental values.

We construct the hydrogen vacancy (V_H_) model by removing one hydrogen atom from the ADP supercell with 48 atoms. Firstly, we compare accuracy of considered electron exchange–correlation functionals on the crystal structure prediction by comparing the calculated the lattice constants of *a*, *b*, and *c* of ADP with experimental data.^50,51^ We then plot the corresponding mean absolute error (in %) in Fig. 1. We can see that HSE03 and PBE0 functionals show larger errors than PBE and HSE06 functionals for both *a* and *c* lattice parameters. PBE and HSE06 functionals show the similar extent on the prediction of lattice parameters. Therefore, we choose PBE functional to optimize the lattice constant and atomic position in order to save the computational cost. The optimized lattice constants by PBE are *a* = *b* = 7.32 Å and *c* = 7.42 Å with deviation of 2.40% and 1.72% with respect to the experimental values of *a* = *b* = 7.50 Å and *c* = 7.55 Å.^44,45^

**Fig. 2:**
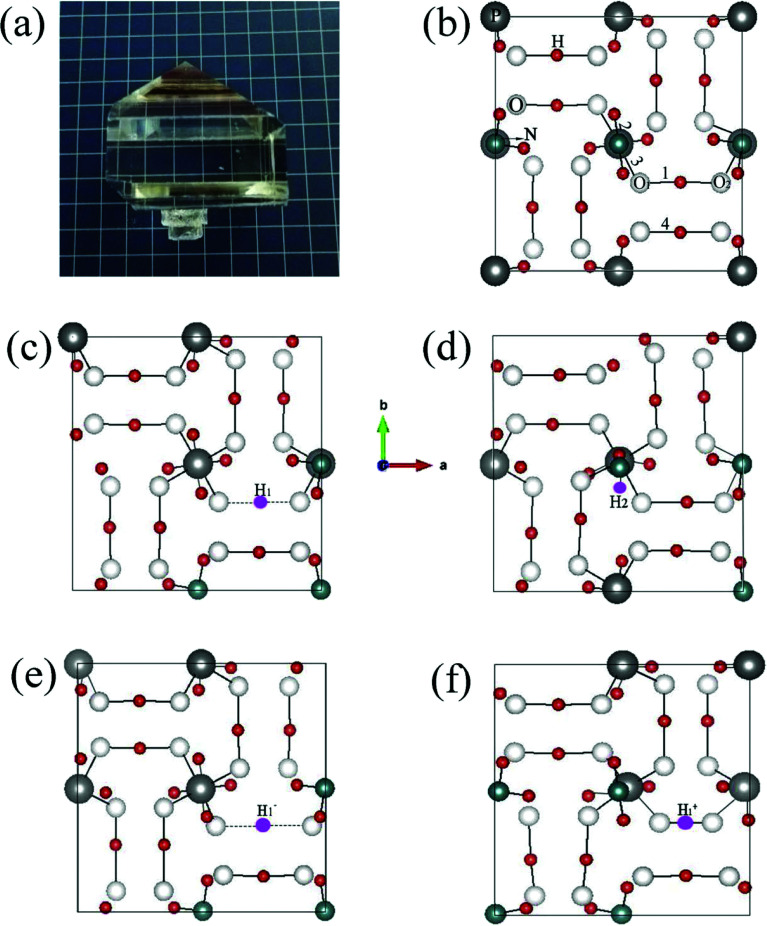
ADP crystal grown with the temperature reduction method (a), top-view of the crystal structures of pristine ADP (b), ADP with neutral H_1_ (c) and H_2_ (d) vacancies, as well as H_1_ vacancies with −1 (e) and +1 (f) charge states. The vacancies are indicated in the small rose red balls.

Fig. 2(a) shows the ADP single crystal grown by the temperature reduction method, and its corresponding crystal structure is shown in Fig. 2(b). The ADP crystal structure is tetragonal with *I*4̄2*d* space group. It is seen from Fig. 2(b) that each NH_4_^+^ group is connected with six (H_2_PO_4_)^−^ groups *via* covalent bond of N–H–O, while each (H_2_PO_4_)^−^ group bonds with four (H_2_PO_4_)^−^ groups by the P–O–H⋯O hydrogen bond.^19^ Therefore, there are two kinds of H atoms in the ADP crystal – H_1_ in the (H_2_PO_4_)^−^ group with the fractional coordinate (0.750, 0.350, 0.375) and H_2_ in the NH_4_^+^ group with the fractional coordinate (0.500, 0.411, 0.937), as labelled in Fig. 2(c) and (d). It is found that V_H_1__ leads to small amount of lattice relaxation whereas V_H_2__ defect causes large atomic distortion regarding to the change of atomic location, bonding length, bonding angle and symmetry of NH_4_^+^ group. In order to accurately obtain the relative stability of V_H_1__ and V_H_2__, we compare the calculated defect formation energies (DFEs) of neutral V_H_1__ and V_H_2__ with different functionals and listed in Table 2. It is seen that although the absolute values of DFEs of V_H_1__ and V_H_2__ varies for different functionals, the DFE of V_H_1__ is always lower than that of V_H_2__ indicating that V_H_1__ is energetically preferable to form in ADP crystal. The easier missing of H_1_ atom is mainly due to the weak hydrogen bond interaction. Therefore, we studied the charged V_H_ defects based on the V_H_1__ model. The model of positively charged 
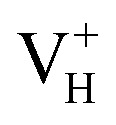
 is constructed by removing one electron from the neutral V_H_ system, and that of negatively charged 
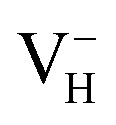
 is constructed by adding one electron to the neutral V_H_ system. The optimized structures are shown in Fig. 2(e) and (f). It is seen that the distance of O_1_ and O_2_ adjacent to the 
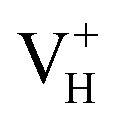
 is dramatically shortened by 37.70% from 2.387 to 1.488 Å (Table 3) as compared to the case of neutral V_H_ defect due to the capture of hole, showing strong interaction of O_1_ and O_2_. In this case, the distance of O_1_–O_2_ is similar to the bond length of 1.49 Å for the neutral peroxyl bridge in vitreous silicon dioxide.^52,53^ The movement of O toward 
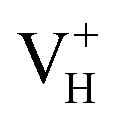
 defect leads to the elongation of P–O bond by 9.08%. On the contrary, the capture of an electron carrier by V_H_ leads to the elongation of the O_1_–O_2_ distance by 20.74% (from 2.387 to 2.882 Å) due to the Coulomb repulsion of captured electron with the electrons around O_1_ and O_2_, indicating a much weak interaction of O_1_ and O_2_. The outward movement of O regarding to 
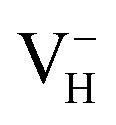
 only leads to the slight shortening of P–O bond by 2.01%, showing small influence on the PO_4_^3−^ group.

**Table 2:** The calculated defect formation energies of neutral V_H_1__ and V_H_2__ with different functionals

Functionals	Defect formation energies *E*_f_ (eV)	
	V_H_1__	V_H_2__
PBE	3.06	3.44
HSE06	4.56	4.70
PBE0	4.37	4.45
HSE03	3.94	4.19

**Table 3:** Bond lengths and distance between atoms closed to the hydrogen vacancy as labelled in Fig. 2. The numbers of chemical bonds are related to those labelled in Fig. 2(b). The units are in Å

Bonds/distance	Pristine ADP	V_H_1__	V_H_2__	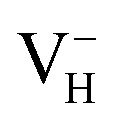	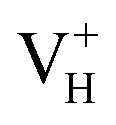
1	1.200	—	1.343	—	—
2	1.039	1.038	—	1.006	1.035
3	1.551	1.542	1.528	1.501	1.682
4	1.200	1.201	1128	1.207	1.195
O_1_–O_2_	2.401	2.387	2.437	2.882	1.488

### 3.2 Relative stability of hydrogen vacancies in ADP

We investigate the relative stability of hydrogen vacancies in ADP crystal by calculating the defect formation energies dependent with the Fermi level and the charge transition levels. At the beginning, we examine the influence of calculation model and parameters on the accuracy of defect formation energies of charged point defects by taking 
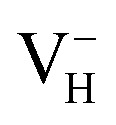
 as an example. The charged defects always introduce finite-size error that arise from the electrostatic interaction between charged defects in neighboring cell images and the structural strain introduced by the defect due to the limited size of the supercell within periodic boundary condition. We therefore first compare the defect formation energies of 
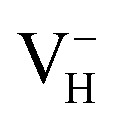
 calculated in 48-atom and 384-atom supercells within PBE functional, as shown in Table 4. It is seen that the calculated defect formation energies of 
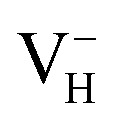
 in 384-atom supercell is only 0.04 eV lower than that in 48-atom supercell, indicating extremely slow energy change with the increase of supercell size until to infinite limit. Therefore, the finite-size error to the defect formation energies of charged V_H_ could be neglected, and the smaller supercells (48 atoms) are used to investigate the energetic and electronic properties of V_H_ in ADP crystal in order to save the computational cost. Second, we consider the influence of vdW interaction on the defect formation energy of 
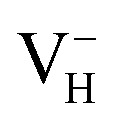
. The inclusion of vdW interaction by DFT/vdW-WF2 method has been proved to obtain more accurate description of the molecule–surface interaction and the system with hydrogen bond interaction.^54–59^ Here we compare the calculated defect formation energies of 
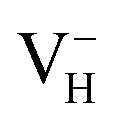
 with and without vdW correction within PBE functional, as shown in Table 4. It is seen that the vdW correction could reduce the defect formation energy of 
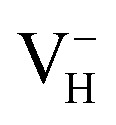
 by 0.24 eV, which is one order of magnitudes larger than the error of finite-size effect. Such energy difference may lead to the different result of the relative stability of charged V_H_ in ADP crystal, and the vdW correction should thus be considered in the following calculations. Finally, we consider the effect of screened exchange potential by comparing the calculated defect formation energies of 
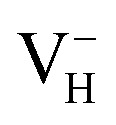
 by PBE and HSE06. From the calculation results shown in Table 4 we can see that the HSE06 functional that mixing 25% nonlocal Hartree–Fock exchange with PBE could largely enhance the defect formation energy of 
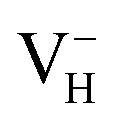
 by 3.35 eV. It is one order of magnitudes larger than the correction of vdW interaction, and should not be neglected. As HSE06 has been proved to better describe the defect formation energy, electron localization, and defect levels than GGA, HSE06 functional is used in the following calculations.^60,61^ Overall, the energetics and electronic structures of V_H_ are calculated within HSE06 hybrid functional with vdW correction by using tetragonal ADP supercells containing 48 atoms after carefully parameter examination.

**Table 4:** The comparison of defect formation energies of V_H_ in ADP when considering different defect location, supercell size, vdW correction, and hybrid electron exchange potential

Parameters	Defect formation energies *E*_f_ (eV)
V_H_1__, HSE06, 48 atoms	4.56
V_H_2__, HSE06, 48 atoms	4.70
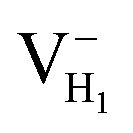 , PBE, 384 atoms	3.16 − *E*_F_
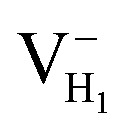 , PBE, 48 atoms	3.12 − *E*_F_
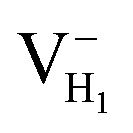 , PBE + vdW, 48 atoms	2.88 − *E*_F_
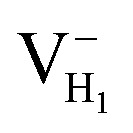 , HSE06 + vdW, 48 atoms	4.33 − *E*_F_

**Fig. 3:**
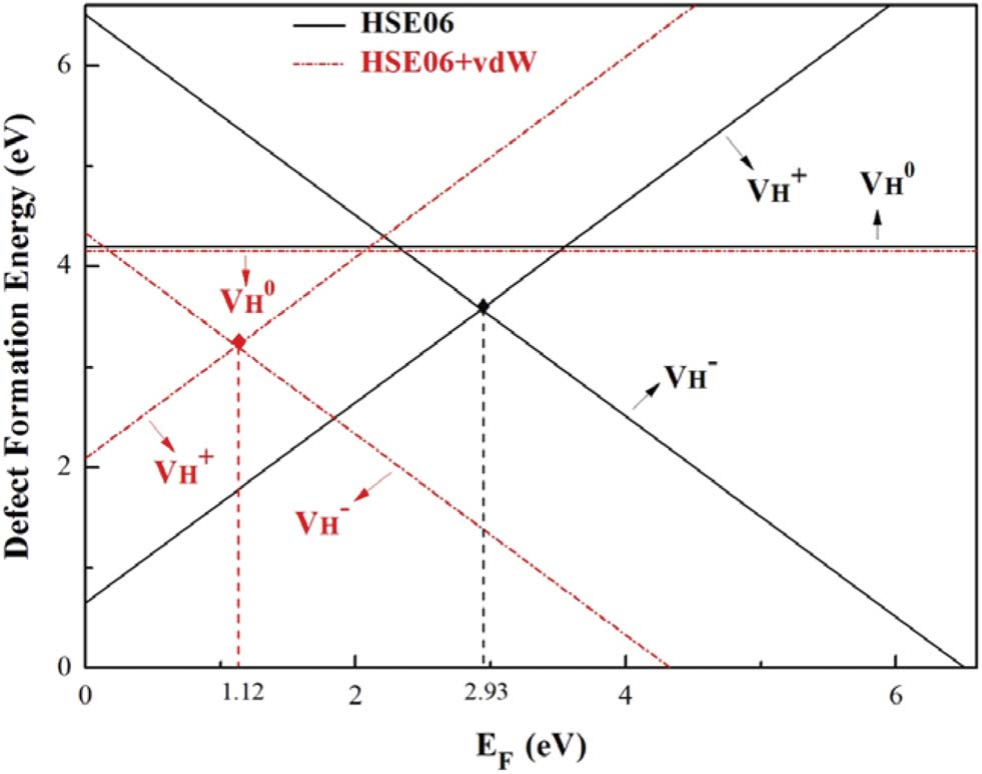
Defect formation energies of V_H_ with positive, neutral, and negative charge states as a function of the Fermi level for HSE06 and HSE06 + vdW methods. The range of Fermi energy corresponds to the calculated fundamental band gap of 6.76 eV for pristine ADP crystal.

The calculated defect formation energies of 
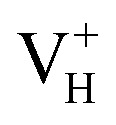
, 
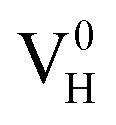
, and 
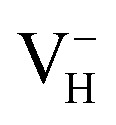
 with and without vdW correction are compared in Fig. 3. We found that the vdW interaction play a key role in the calculation of defect formation energies and charge transition levels. For example, the vdW correction largely shifts the charge transition level *ε*(+/−) toward the valence band maximum (VBM) from 2.93 eV to 1.12 eV. We could get different conclusions according to this result: without the consideration of vdW interaction, the positively charged hydrogen vacancy 
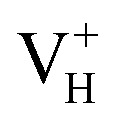
 is the most stable defect in majority of ADP crystals, and the negatively charged hydrogen vacancy 
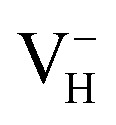
 only exists in near-stoichiometric crystal; whereas 
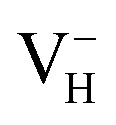
 is more stable than 
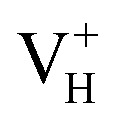
 when the Fermi level moves up to 1.12 eV if the vdW interaction is considered. The calculation results with vdW interaction are for sure more accurate than those without the vdW interaction. We thus only discuss the calculation results with vdW interaction. It is seen that 
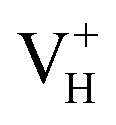
 is more stable than 
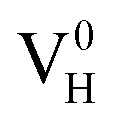
 and 
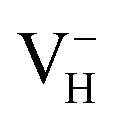
 when the Fermi level lies near the VBM while 
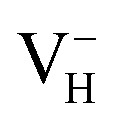
 is energetically preferable when the Fermi level lies higher than 1.12 eV. As in ADP crystals, H atoms are much easier to be missed than N, P, and O atoms to form V_H_ defects, the Fermi level could lies below the middle of the electronic band gap of ADP. Therefore, in this region, 
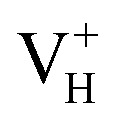
 and 
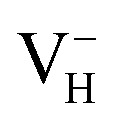
 are all the possible defects in practical ADP crystals. The results demonstrate that the neutral 
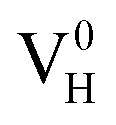
 is instable in the ADP crystal, which is similar to the case in SiO_2_.^62^ The instability of the neutral 
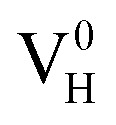
 defect indicates that the hydrogen vacancies in majority of ADP crystals prefer to trap hole or electron carriers. There are two extreme situations: when the defect concentration of hydrogen vacancies is high, V_H_ defects prefer to capture hole carriers and act as acceptor centers; if the grown ADP crystal is with good quality (very small concentration of V_H_), these hydrogen vacancies mainly prefer to capture electron carriers and act as donor centers. If the situation is in between, part of V_H_ may capture holes and the rest captures electrons which results in the spatial charge separation in ADP crystals. These results may give a reasonable explanation regarding to the experimentally observed inflection point in the conductivity curve from the aspect of defect behavior.^63^ At the low temperature region, the concentration of hydrogen vacancies is very low, and the amount of the electron carriers in the crystals is thus also small. In this case, 
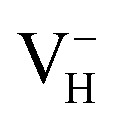
 is energetically preferable and therefore, the neutral hydrogen vacancy defects prefer to capture electrons to form stable 
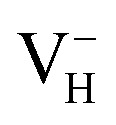
 defects. It further reduces the concentration of electron carriers, and mainly contributes to the low conductivity of ADP crystal. With the increase of temperature, more hydrogen atoms could escape from their original lattice sites due to the thermal vibration, leaving a large amount of hydrogen vacancies in the lattice. On the one hand, it will largely increase the concentration of electron carriers in the crystal. On the other hand, with the increase of hydrogen vacancy concentration, a large part of the vacancy defects prefer to capture hole carriers instead of electron carriers to form stable 
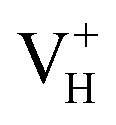
 defects, which leaves much more electrons in the crystal, leading to the sharp increase of the conductivity of the ADP crystal. The well explanation of the measured conductivity phenomenon further proved the importance of considering the vdW interaction in ADP calculations.

### 3.3 Electronic structure of hydrogen vacancies in ADP

In order to better understand the electron/hole trapping behaviors and the defect induced impurity states in the band gap of ADP crystal, we calculated the density of states of stable hydrogen vacancies and the electron distributions around the hydrogen vacancy defects in ADP crystal. First, we compare the calculated electronic band gaps of ADP by different functionals. As shown in Table 5, HSE06 functional shows the best description of band gap of ADP crystal (6.76 eV) as compared to the experiment value 6.96 eV,^64^ indicating that HSE06 could well reproduce the electronic properties such as density of states, electron distribution of this system. The local PBE functional could underestimate the band gap as usual, and the PBE0 functional is proved to overestimate the band gap compared with HSE06, which has been reported in the literature.^50,51^ The HSE03 functional shows fewer consistence with experiment than HSE06. Also, we found that the consideration of vdW correction could not change the calculated band gap of ADP, showing little influence on the electronic structures of ADP crystal.

**Table 5:** The band gap of the pristine ADP crystal with different DFT functionals

Functionals	The band gap (eV)
Experimental	6.96
PBE	5.97
HSE06	6.76
PBE0	7.79
HSE03	6.51

**Fig. 4:**
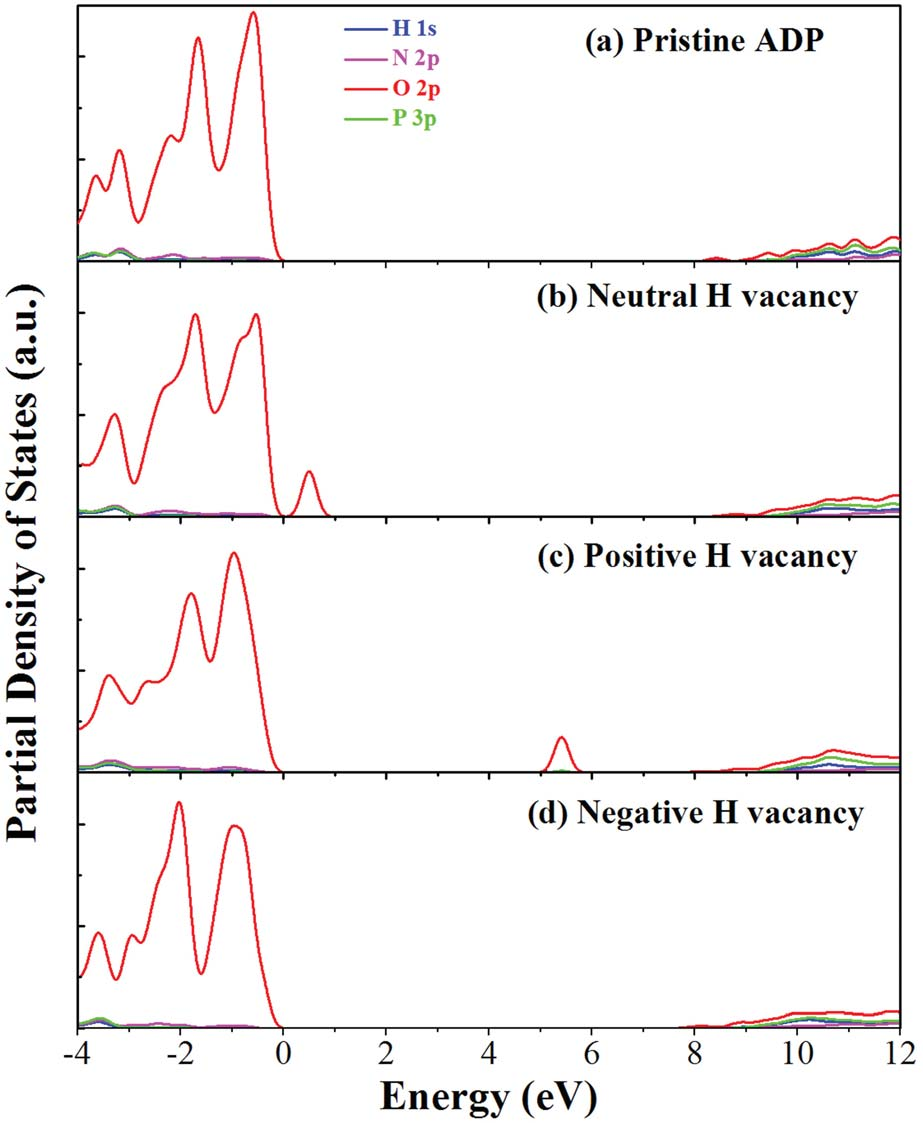
PDOS of H, N, O, and P in pristine ADP (a) and crystal with neutral (b), positively (c) and (d) negatively charged hydrogen vacancies.

**Fig. 5:**
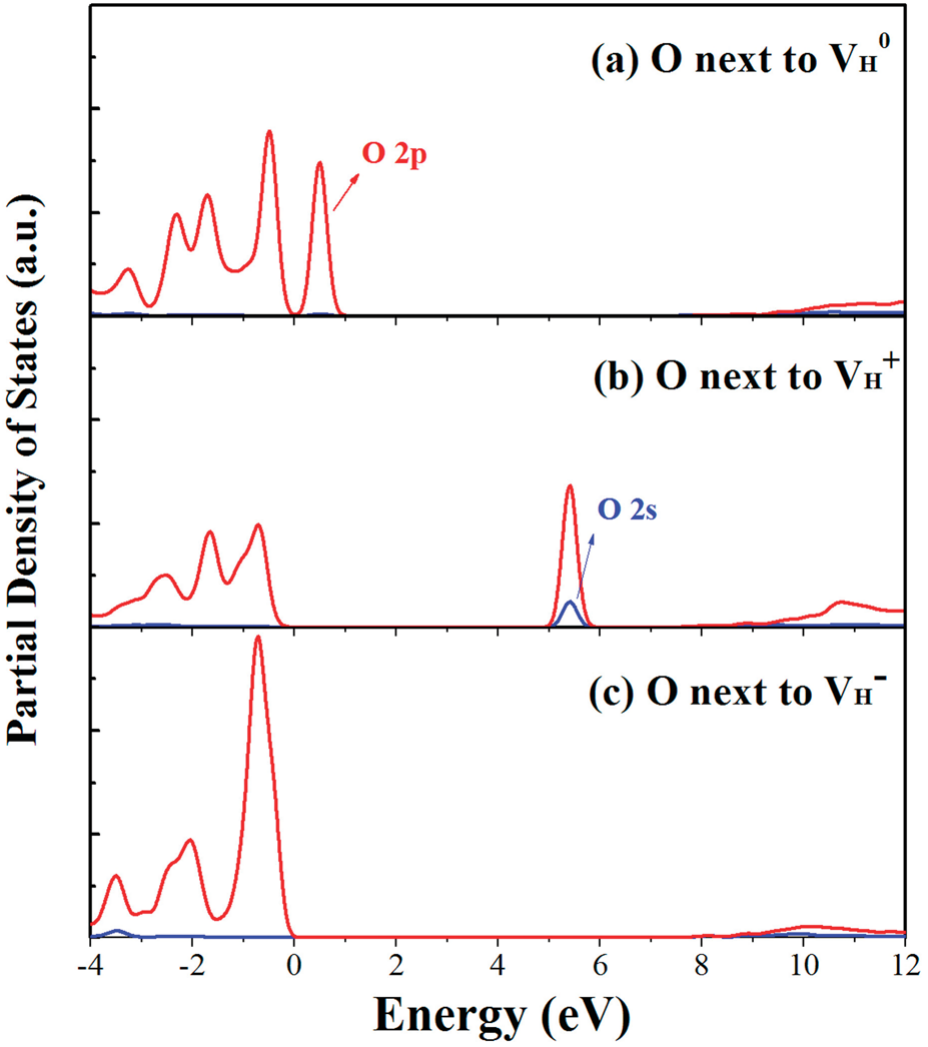
PDOS of the O atoms adjacent to the neutral (a), positive (b) and negative (c) H vacancies.

The calculated partial density of states (PDOS) of neutral, positively and negatively charged hydrogen vacancies as compared to those of pristine ADP crystal are shown in Fig. 4. It is seen that the VBM of pristine ADP is mainly derived from O 2p states while the conduction band minimum (CBM) comes from the mixing of H 1s, O 2p, and P 3p states. The formation of a neutral 
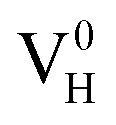
 defect introduces a half-occupied defect state at about 0.5 eV above the VBM in the band gap (Fig. 4b). This defect state is derived from O 2p states of its neighboring O atoms (Fig. 5a), which is similar to the results of electronic properties calculations for hydrogen vacancy in silica.^62^ It could introduce weak optical absorption just near the absorption edge of pristine ADP crystal that comes from the electron transition from the defect state to the CBM and may hardly be recognized.

**Fig. 6:**
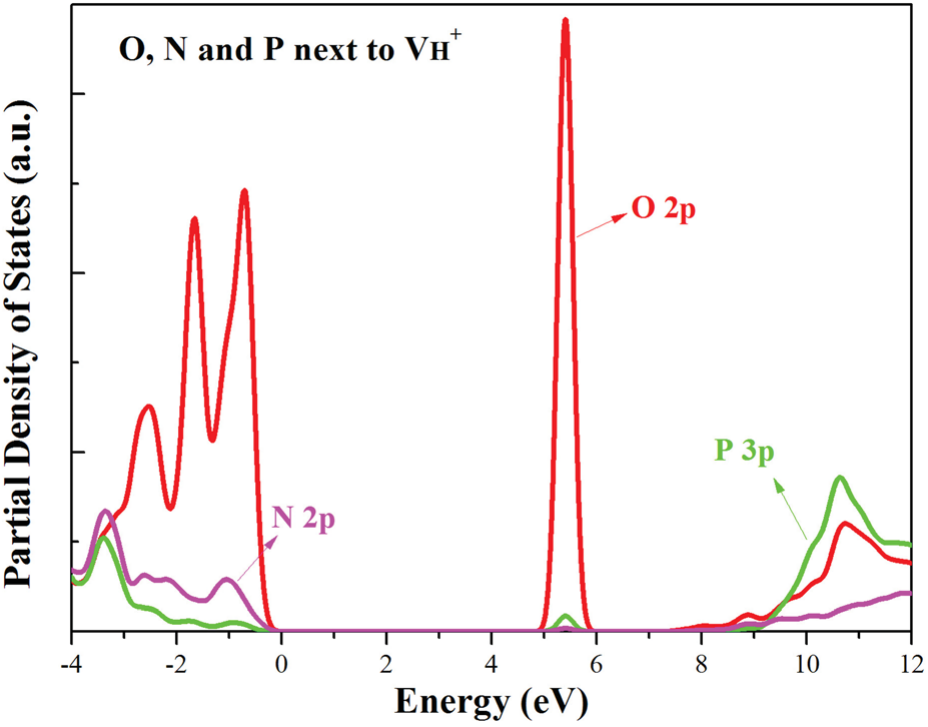
PDOS of the O, N and P atoms adjacent to the positive H vacancy.

**Fig. 7:**
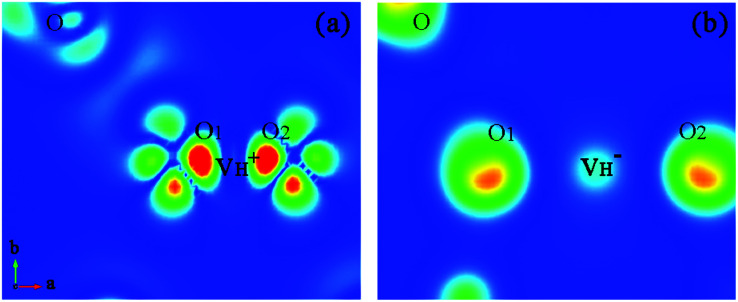
The charge density difference contour maps for 
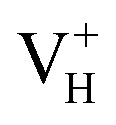
 and 
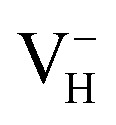
. The regions from blue to red correspond to the electron density from 0 to 0.17 e Å^−3^.

When the neutral V_H_ captures one hole to form 
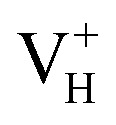
 defect, the defect state in the band gap shifts toward CBM by 4.7 eV and the band gap is reduced to 5.2 eV. This defect leads to the stronger coupling interaction of its adjacent O atoms, and finally forms O–O peroxyl bridge which is similar to the phenomenon of the positive hydrogen vacancy in KDP and the oxygen atom in a peroxyl bridge in silica.^52,65^ Finally, the hydrogen vacancy could capture a hole with its two adjacent O atoms to form a molecular-type polaron. The contributions of the adjacent O atoms and the other adjacent atoms have been plotted in Fig. 5b and 6 respectively. We can see that the defect state of 
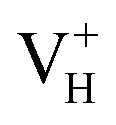
 comes from the mixing of 2s and 2p states of its two neighboring O atoms that could also be confirmed from the charge density difference plot in Fig. 7a. In the above discussion, we found that the P–O bond is stretched and the PO_4_^3−^ group also moves toward the 
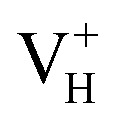
 due to the formation of O–O peroxyl bridge. Therefore, there is also a small amount of contribution of P 3p state on the 
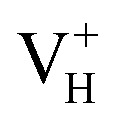
 defect state in the band gap (Fig. 6). The VBM is mainly from the 2p states of the adjacent O atoms.

Another case is that the neutral V_H_ defect captures one electron to form 
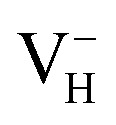
 defect. In this case, the isolated defect state in the band gap is full-occupied and downshifted to the valence band. At the same time, the valence bands are slightly upshifted due to the Coulomb repulsion of electrons. Therefore, the band gap of 
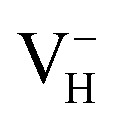
 defect is reduced to 6.27 eV, and the contribution of adjacent O 2p states to the VBM becomes much stronger (Fig. 5c). As compared to the 
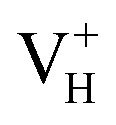
 defect, the interaction between O_1_ and O_2_ becomes much weaker, while that of P and O becomes stronger. It could not introduce any new optical absorption for this defect.

By analyzing the electronic structures of the hydrogen vacancies, we get that only the positive charged hydrogen vacancy 
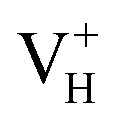
 is stable and could introduce additional optical absorption with respect to the intrinsic optical absorption. According to the calculation results, the new optical absorption introduced by 
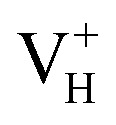
 could appear at about 260 nm. The meaning of the stability of 
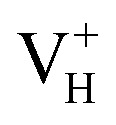
 is that when the defect concentration is high, the additional optical absorption would be strengthened and easily detected by experimental measurement.^66^ They could also induce the precursor to absorb the laser energy, leading to the material damage and thus decreasing the laser damage threshold of ADP. Therefore, the concentration of positively charged hydrogen vacancy defects is suggested to be largely reduced during the growth of the crystal. In our experiment, we also observe the optical absorption at 355 nm (3.49 eV) that has been detected in many other experiments, such as ref. 3. It is not expected to come from the intrinsic H vacancy as the calculated transition energy is not consistent with the measured absorption energy (more than 1 eV difference). As we have tested the validity of local PBE functional and many types of hybrid functionals on the description of crystal structure and electronic properties of ADP crystal, the large energy difference of 1 eV could not be from the calculation error. We infer that the optical absorption at 355 nm could come from the extrinsic impurity centers in ADP crystal, which should be intensively investigated later.

## 4. Conclusions

In conclusion, the density functional theory with (semi)local GGA and hybrid HSE06 exchange–correlation functionals were carried out to investigate the structures, relative stability, and electronic structures of the neutral and charged hydrogen vacancies in ADP crystals. The influence of supercell size, van der Waals interaction, and the screened exchange potential on the defect formation energies of charged hydrogen vacancies is carefully examined firstly. The calculation results show that the van der Waals interaction and the screened exchange potential have major effect on the calculation accuracy of both energetics and electronic structures, and thus should be considered. By comparing the defect formation energies of neutral V_H_ in (H_2_PO_4_)^−^ and NH_4_^+^ groups, we found that the H atom in the (H_2_PO_4_)^−^ group is easier to be missed to form V_H_ defect. However, the neutral V_H_ is unstable in the ADP crystals. It could capture a hole carrier to form its positive charge state or capture an electron to form its negative charge state in majority of ADP crystals. For the 
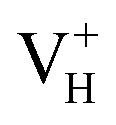
 defect, the hole is trapped around the two O atoms adjacent to the hydrogen vacancy to form a molecular-type polaron. We infer that the transition of 
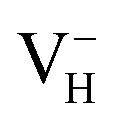
 to 
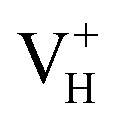
 with the increase of temperature may be the dominant reason for the break point of the conductivity of ADP crystal in the high temperature region. On the other hand, 
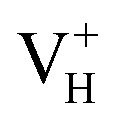
 defect introduces a deep defect state in the middle of band gap. It could act as an optical absorption center in experiment and lead to the special optical absorption phenomenon and optical damage under irradiation by high-intensity laser beam.

## Conflicts of interest

There are no conflicts to declare.

## Supplementary Material
